# Impact of malnutrition on postoperative delirium development after on pump coronary artery bypass grafting

**DOI:** 10.1186/s13019-015-0278-x

**Published:** 2015-05-20

**Authors:** Donata Ringaitienė, Dalia Gineitytė, Vaidas Vicka, Tadas Žvirblis, Jūratė Šipylaitė, Algimantas Irnius, Juozas Ivaškevičius, Tomas Kačergius

**Affiliations:** 1 Clinic of Anesthesiology and Intensive care, Vilnius University, Faculty of medicine, Vilnius, Lithuania; 2 Vilnius University, Faculty of Medicine, Vilnius, Lithuania; 3 Hematology, Oncology and Transfusion Medicine Center, Vilnius University Hospital Santariskiu Klinikos, Vilnius, Lithuania; 4 Hepatology and Gastroenterology Department, Vilnius University faculty of Medicine. Centre of Hepatology, Gastroenterology and Dietetics, Vilnius University Hospital Santariskiu Klinikos, Vilnius, Lithuania; 5 Center of Anesthesia and Intensive care Vilnius University Hospital Santariskiu Klinikos, Santariskiu 2, Vilnius, Lt 08448 Lithuania; 6 Department of Physiology, Biochemistry, Microbiology and Laboratory Medicine, Faculty of Medicine, Vilnius University, Vilnius, Lithuania

**Keywords:** Malnutrition, Cardiac surgery, Delirium, Postoperative outcomes

## Abstract

**Background & aims:**

Even though malnutrition is frequently observed in cardiac population outcome data after cardiac surgery in malnourished patients is very rare. No thorough research was done concerning the impact of malnutrition on neuropsychological outcomes after cardiac surgery. The aim of our study was to analyze the incidence of postoperative delirium development in malnourished patients undergoing on pump bypass grafting.

**Methods:**

We performed a cohort study of adults admitted to Vilnius University Hospital Santariskiu Clinics for elective coronary artery bypass grafting. The nutritional status of the patients was assessed by Nutritional Risk Screening 2002 (NRS-2002) questionnaire the day before surgery. Patients were considered as having no risk of malnutrition when NRS-2002 score was less than 3 and at risk of malnutrition when NRS-2002 score was ≥3. During ICU stay patients were screened for postoperative delirium development using the CAM-ICU method. and divided into two groups: delirium and non delirium. The statistical analysis was preformed to evaluate the differences between the two independent groups. The logistic regression model was used to evaluate the potential preoperative and intraoperative risk factors of postoperative delirium.

**Results:**

Ninety-nine patients were enrolled in the study. Preoperative risk of malnutrition was detected in 24 % (n = 24) of the patients. The incidence of early postoperative delirium in overall study population was 8.0 % (n = 8). The incidence of the patients at risk of malnutrition was significantly higher in the delirium group (5 (62.5 %) vs 19 (20.9 %), *p* <0.0191). In multivariate logistic regression analysis risk of malnutrition defined by NRS 2002 was an independent preoperative and intraoperative risk factor of postoperative delirium after coronary artery bypass grafting (OR: 6.316, 95 % CI: 1.384-28.819 *p* = 0.0173).

**Conclusions:**

Preoperative malnutrition is common in patients undergoing elective coronary artery bypass grafting. Nutrition deprivation is associated with early postoperative delirium after on pump coronary artery bypass grafting.

## Background

Preoperative loss of body weight can be found in approximately 10 %–25 % of patients undergoing cardiac surgery [1–3]. A decrease in body mass index is prevalent in the older population, particularly among institutionalized elders and those with chronic disease.

Heart failure and cachexia often co-exist. Body composition alterations that occur in heart failure is complex phenomenon that involves the interplay of numerous factors including food intake, absorption, immunological and neurohormonal activation, and the balance between catabolic and anabolic states. Studies report that cachexia had a incidence of 10 % in patients with New York Heart Association (NYHA) class III or IV and the prevalence of 12 %–15 % in NYHA II to IV [4, 5].

Patients undergoing cardiac surgery are often prone to a number of co-morbidities despite impaired heart function. Surgical stress increases their susceptibility to accelerated weight loss. Observational studies reveal that up to 40 % of the critically ill patients need nutritional support preoperativelly [6, 7].

This combination of multiple physiological insults caused by chronic disease and procedure itself can result in a substantial physical and functional decline of the nutritional status.

Nutrition is essential for all the functions of the body. Emerging evidence proves that deficiencies of certain nutrients might be playing an important role in the development of severe cognitive problems [8]. Micronutrients and other vitamin deficiencies such as niacin or thiamine are known to have an impact on delirium onset due to impaired neurotransmission. Decreased plasma protein is associated with elevated serum free drug concentrations that are normally protein bound.

Data on preoperative malnutrition in relation to clinical outcomes after cardiac surgery is limited. Therefore we aimed to analyze the associations between malnutrition and postoperative delirium in a sample of patients undergoing cardiac surgery. We hypothesized that there is a positive relationship between preoperative risk of malnutrition and postoperative mental status alterations.

## Methods

### Patients

A prospective cohort study was conducted in a tertiary referral university hospital between April and June 2012.

Ninety-nine consecutive patients undergoing elective on pump coronary artery bypass grafting were screened to enroll in the study. The follow-up period of all the patients was limited to ICU stay.

This study was approved by the research ethics committee of the clinics and informed consent was obtained from all of the patients.

Upon admission that is one day before the surgery all of the patients were assessed for nutritional risk with the following tools: Nutritional Risk Screening 2002 (NRS-2002) questionnaire, anthropometric measurements and laboratory data. The nutritional assessments were performed by trained physicians.

Preoperative evaluation, premedication, anesthesia and surgery were performed according to the institutional protocols; no adjustments were made for study participants.

Anesthesia induction consisted of administration of midazolam, fentanyl and remifentanil. Anesthesia was maintained by continuous infusion of fentanyl or remifentanil incombination with propofol infusion initiated and continued during cardiopulmonary bypass. Cardiopulmonary bypass was performed according to standard protocol: mean arterial pressure was kept >60 mmHg, blood flow was maintained at 2.4 l/min/m^2^ of body surface area. Myocardial protection during aortic cross-clamping period was achieved with minimally diluted tepid blood cardioplegia.

Saphenous veins, radial artery and internal mammary artery were used as conduits for myocardial revascularization. Proximal anastomoses were performed with partial occluding aortic clamp.

After surgery patients were admitted directly to the ICU and weaned off mechanical ventilation according to the standard respiratory care protocol Following criteria were used to extubate the patient: patient is awake, body core temperature is >36,8 C, patient is haemodynamically stable or requires low doses of inotropic support, drainage from the wound is less than 100 ml/h for at least two hours, no shivering, adequate diuresis present, spontaneous breathing measurements of PaO2 >80 mmHg and PaCO2 <49 mmHg.

Postoperative analgesia was achieved using opioid analgesics administered intravenously combined with non-steroidal anti-inflammatory drugs. After extubation analgesic regiment was changed by the attending physician using the Visual Analog Scale (VAS). Infusion rates of propofol and midazolam used for sedation were titrated in order to achieve and maintain a Ramsay Sedation Score 7 (RSS7) of 3 before extubation and of 2 after extubation. Patients were weaned off propofol or midazolam infusions before extubation. If a patient developed delirium treatment with haloperidol 5 mg every 2–4 h was used as needed. Haloperidol was used only after a diagnosis of delirium was established. All clinical decisions regarding the time of extubation and administration of medications were made on the basis of the standardized protocols and clinical judgment of the treating ICU physician.

### Nutritional risk screening

The NRS-2002 was introduced by the European Society of Parenteral and Enteral Nutrition as the method forscreening and assessing the nutritional status of hospitalized patients [9].

Its stated purpose was “Identification of those hospitalized patients, who are malnourished or at risk for malnourishment and who would gain benefit from the improvement of their nutritional situation”. The NRS-2002 consists of an evaluation of impaired nutritional status and severity of disease and an age adjustment for patients aged >70 years (Table 1).

**Table 1:** Nutritional risk screening scale (NRS-2002)

Initial screning			
1	Is Bmi <20.5?	Yes	No
2	Has the patient lost weight within the last 3 months?		
3	Has the patient had a reduced dietary intake in the last week?		
4	Is the patient severed ill? (e.g. in intensive therapy)		
Yes: if the answer is ‘Yes’ to any question, the screening in Table 2 is performed. No: if the answer is ‘No’ to all question, the patient is rescreened at weekly intervals. If the patient e.g. is schedule for a major operation, a preventive nutritional care plan is considered to avoid the associated risk status.			
Final screening			
Impaired nutritional status	Severity of disease (≈ increase in requirement)		
Absent Score 0	Normal nutritional status	Absent Score 0	Normal nutritional requirements
Mild Score 1	Wt loss >5 % in 3 mths or Food intake below 50–75 % of normal requirement in preceing week	Mild Score 1	Hip fractured, Chronic patients, in particular with acute complications: cirrhosis, COPD. *Chronic hemodialysis, diabetes, oncology*
Moderate Score 2	Wt loss >5 % in 1 mth (>15 % in 3 mths) or BMI 18.5-20.5 + impaired general condition or Food intake 25-60 % of normal requirement in preceding week	Moderate Score 2	Major abdominal surgery, Stroke *Severe pnemonia, hematologic maligancy*
Severe Score 3	Wt loss >5 % in 1 mth (>15 % in 3 mths) or BMI <18.5+ impaired general condition or Food intake 0-25 % of normal requirement in preceding week in preceding week.	Severe Score 3	Head injury, Bone marrow transplantation. *Intensive care patients (APACHE >10).*
Score	+	Score	-Total
Age	if ≥70 years: add 1 to total score above	- age-adjusted total score	
Score ≥3: the patient is nutritionally at-risk and a nutritional care plan is initiated; Score <3: weekly rescreening of the patient. If the patient e.g. is schedule for a major operation, a preventive nutritional care plan is considered to avoid the associated risk status.			

Patients were considered as being of no risk of malnutrition when NRS-2002 score was less than 3 and at risk of malnutrition, when NRS-2002 score was ≥3.

### Delirium screening

Diagnosis of delirium was made using Confusion Assessment Method (CAM) for Intensive Care Unit [10]. The evaluation is based on acute change in mental status with a fluctuating course, inattention, and either disorganized thinking or an abnormal level of consciousness. Delirium screening was started 24 h past surgery and repeated every 8 h during the patients’ ICU stay.

### Assessment of risk factors

Preoperative and intraoperative variables expected to be associated with development of postoperative delirium were obtained prospectively from a preoperative interview of the patient and using chart records. Risk factors listed in further analysis Table 2 are defined as: chronic lung disease - long term use of bronchodilators or steroids for lung disease; peripheral vascular disease - one or more of the following: claudication, carotid occlusion or >50 % stenosis, amputation because of arterial disease, previous or planned intervention on the abdominal aorta, limb arteries or carotids; advanced age was defined as documented age of more than 60 years, pre-operative renal failure was defined as preoperative serum creatinine level of 2.0 mg/dl or more. Hypertension was defined as patients receiving treatment for hypertension or high blood pressure (*i.e.* >140 mmHg systolic or >90 mm diastolic) noted during preoperative stay.

**Table 2:** Comparison of preoperative and operative variables of patients in delirium and non delirium groups

Variable	Delirium, N = 8	Non delirium, n = 91	*P*-value
Preoperative and Demographic variables			
Gender, n (%)			0.6891
Male	5 (62.5)	65 (71)	
Female	3 (37.5)	26 (29)	
Age, years (mean ± SD)	69.9 ± 6.3	67.4 ± 7.9	0.5802
Weight, kg (mean ± SD)	92.6 ± 15.9	84.7 ± 13.4	0.2523
Height,cm (mean ± SD)	168.6 ± 10.7	169.7 ± 8.1	0.7429
EuroScore (mean ± SD)	2 ± 1	2 ± 1.5	0.7635
NYHA >2 (n (%))			1.000
1–2	0 (0)	8 (15)	
>2	8 (100)	78 (85)	
LVEF, %(mean ± SD)	46.3 ± 7.4	50.2 ± 6.8	0.1602
STS predicted mortality	3.2 ± 3.4	1.8 ± 1.9	0.2030
STS predicted morbidity/mortality	25.3 ± 21.2	15.7 ± 10.5	0.2202
Malnutrition (NRS 2002), n (%)			0.0191
Absent	3 (37.5)	72 (79.1)	
Present	5 (62.5)	19 (20.9)	
Smoking history,n (%)			0.6759
Absent	6 (75)	72 (79)	
Present	2 (25)	19 (21)	
Alcohol abuse,n (%)			1.0000
Absent	8 (100)	88 (97)	
Present	0 (0)	3 (3)	
Diabetes,n (%)			0.4126
Absent	5 (62.5)	69 (76)	
Present	3 (37.5)	22 (24)	
Peripheral vascular disease,n (%)			0.3832
Absent	5 (62.5)	71 (78)	
Present	3 (37.5)	20 (22)	
Hypertension,n (%)			1.000
Absent	0 (0)	4 (4)	
Present	8 (100)	87 (96)	
COPD,n (%)			1.000
Absent	8 (100)	84 (92)	
Present	0 (0)	7 (8)	
Renalfailure,n (%)			1.000
Absent	8 (100)	89 (98)	
Present	0 (0)	2 (2)	
MI,n (%)			1.000
Absent	4 (50)	40 (44)	
Present	4 (50)	51 (56)	
MI <30 days,n (%)			0.6806
Absent	5 (62.5)	66 (74)	
Present	3 (37.5)	24 (26)	
Preoperative Hb, g/l (mean ± SD)	127.8 ± 7.8	137.1 ± 14.9	0.0442
Operative values			
Surgery time, min. (mean ± SD)	223.1 ± 41.6	217.7 ± 46.3	0.7188
CPB time,min. (mean ± SD)	103.0 ± 19.7	103.1 ± 29.9	0.8865
Ao cross clamp time, min. (mean ± SD)	60.8 ± 11.4	66.3 ± 19.2	0.2982

### Statistical analysis

Descriptive statistics were used to describe baseline characteristics. Patients were divided in two groups: delirium and non delirium. Mann–Whitney-*U* test was used to evaluate the differences between the two independent groups. The differences between independent two qualitative data groups were evaluated by Fisher exact test. Univariate and multivariate logistic regression models were used to evaluate the potential preoperative and intraoperative risk factors for postoperative delirium. Factors found to be significant in the univariate logistic regression analysis were further entered into multivariate logistic regression model using forward model selection process. A two-tailed *p*-value less than 0.05 was considered to be significant. Statistical analysis was performed using Statistical Analysis System (SAS) package version 9.2.

## Results

There were 70 (71 %) males and 29 (29 %) females and the mean age of the cohort was 67.6 years (range 50–83 years).

After applying the nutrition risk assessment scale NRS-2002, 24 % (n = 24) of the patients were considered at risk of malnutrition.

Intensive care unit delirium was detected in 8 % of patients during the early postoperative period.

Comparing demographic and preoperative variables were compared, Patients in the delirium and non-delirium groups were subjected to same incidence of co-morbidities such as arterial hypertension, diabetes or chronic obstructive pulmonary disease (Table 2).

Both groups had similar Society of Thoracic Surgeons (STS) risk predicted morbidity or mortality rate (Table 2).

The incidence of patients at risk of malnutrition was significantly higher in delirium group (5 (62.5 %) vs. 19 (20.9 %), *p* <0.0191). Preoperative laboratory values were similar in both groups, except preoperative hemoglobin level, which was significantly lower in delirium group (127.8 ± 7.8 vs. 137.1 ± 14.9, *p* = 0.0442). There were no statistically relevant differences comparing the duration of surgery or aortic cross clamp time (Table 2).

In multivariate logistic regression analysis, NRS 2002 defined risk of malnutrition was an independent preoperative and intraoperative risk factor of postoperative delirium development after coronary artery bypass grafting (OR: 6.316, 95 % CI:1.384–28.819). Univariate and multivariate logistic regression analysis is shown in Table 3.

**Table 3:** Univaried and multivariate analysis of delirium predictors after coronary artery bypass grafting

Factor	Odds ratio		*P*-value			*P*-value
	Estimate	95 % CI		Estimate	95 % CI	
	Univariate			Multivariate		
Preoperative						
Gender	0.667	0.148-2993	0.5967	n.i.		
Older age	1.045	0.945-1.155	0.3938	n.i.		
Higher weight	1.043	0.988-1.101	0.1240	n.i.		
NYHA (2 vs. 3)	<0.001	<0.001-999.999	0.9687	n.i.		
NYHA (2 vs. 4)	<0.001	<0.001-999.999	1.0000	n.i.		
MI	0.784	0.185-3332	0.7420	n.i.		
MI <30 days	1.650	0.366-7.437	0.5145	n.i.		
Renal failure	<0.001	<0.001-999.999	0.9857	n.i.		
COPD	<0.001	<0.001-999.999	0.9732	n.i.		
Hypertension	>999.999	<0.001-999.999	0.9798	n.i.		
Periferial vascular disease	2.130	0.486-9.689	0.3279	n.i.		
Diabetes	1.882	0.416-8516	0.4117	n.i.		
Malnutrition (NRS2003)	6.316	1.384-28.819	0.0173	6.316	1.384-28.819	0.0173
Smoking history	1.263	0.236-6.766	0.7849	n.i.		
Alcohol abuse	<0.001	<0.001- > 999.999	0.0890	n.i.		
Hb	0.957	0.909-1.007	0.0890	n.i.		
Operative risk						
Euroscorell	0.999	0.597-1.672	0.9976	n.i.		
STS predicted mortality	1.237	0.976-1.568	0.0792	n.i.		
STS predicted mortality/mobidity	1.047	1.001-1.095	0.0465	n.s.		
Operative value						
Surgery time	1.002	0.988-1.018	0.7462	n.i.		
CPB time	1.000	0.975-1.025	0.9909	n.i.		
Ao cross clamp time	0.984	0.947-1.023	0.4169	n.i.		

n.i. - not included; n.s. - not significant

## Discussion

Improvement of medical care and application of new therapeutic approaches lead to a change in the patient population. More patients admitted to cardiac surgery are elderly with increased prevalence of chronic diseases. Chronically ill patients often prone to metabolic disorders, low calorie consumption, impaired nutrition and concomitant cerebrovascular disease followed by dementia or depression leading to impaired nutritional intake [11, 12].

The significance of association between declining nutritional status and postoperative delirium in cardiac population is poorly outlined in literature, despite growing interest addressing malnutrition and surgical outcomes [13]. Remarkably, though not considered being at risk of malnutrition, 24 % of elective heart surgery patients in our study experienced nutritional deprivation before CABG surgery. Furthermore, almost every second patient noted a reduction in food intake due to progressing heart disease and preoperative stress.

Evidence suggests that under-nutrition defined as low body mass index (BMI) and protein malnutrition negatively influence quality of life and postoperative outcomes [14, 15].

Our study proved that preoperative malnutrition defined as NRS-2002 ≥3 is independently associated with postoperative mental status alterations during ICU stay. It is worth mentioning that not weight loss per se or height derived BMI being most common quantitative anthropometric measures but complex malnutrition evaluation had predictive value concerning postoperative delirium development. Furthermore, even ESPEN recommended NRS-2002 tools for screening of malnutrition accuracy is being criticized [16]. It is stated that due to congestive heart failure in cardiac surgery population free fluid accumulates in tissues and affects measurements of the body mass and its derivatives, namely BMI. This means that the first two questions in initial screening in NRS-2002 may be compromised. Accounting for these assumptions would increase the accuracy of malnutrition screening and more thoroughly analyze the impact on delirium development after cardiac surgery.

Comparison of the present results with literature data is limited, as only a few studies have analyzed the neurocognitive status in malnourished patients after surgery.

### Study limitations

Neurocognitive decline after cardiac surgery is a complex phenomenon including peri-operative inflammatory response, fluctuating haemodynamics and exposure to anesthesia medications. In present study we evaluated the prognostic weight of malnutrition amongst other preoperative variables, not taking into account the impact of postoperative delirium risk factors. One may argue that having a worse clinical course after the surgery could have affected the neurocognitive status of the patient [17]. Furthermore it should be taken into consideration that delirium group patients had a higher risk of predicted mortality defined by STS preoperatively. However, testing the components of the STS motel and their potency to predict the postoperative delirium did not yield statistically significant results, suggesting even more complex nature of the delirium after surgery.

The major limitation of the study is a small sample size. Having a bigger study population would have allowed to compare the results within the subpopulations and isolate the effect of malnutrition on postoperative delirium. In this pilot study we thrive to emphasize the importance of complex preoperative evaluation of chronically ill heart surgery patients. Even though having a small study size the Preoperative nutritional status still has an important predictive role regarding postoperative delirium development.

Another limitation of the study is the lack of thorough and comprehensive evaluation of preoperative psycho-emotional status of the patients and the cognitive status. Patients were considered psychiatrically healthy if it was not stated otherwise in medical records. Records on preoperative substance abuse and use of psychotropic medications were based on subjective interview with the patient. For further studies objective psychiatric and cognitive evaluation should be included into the protocol.

## Conclusions

Nutritional depletion is a common problem in heart surgery patients prevalent in one fourth of the patients administered for elective heart surgery. Being at risk of malnutrition is an independent predictor of postoperative delirium development.

Nutritional screening before surgery would enable identification of malnourished patients in the beginning of the care and allow initiation of nutritional support. Further research is needed to better understand whether early nutrition support can improve survival after heart surgery.

## Abbreviations

Ao: Aorta
COPD: Chronic obstructive pulmonary disease
CAM – ICU: Confusion assessment method for intensive care
CPB: Cardiopulmonary bypass
EuroScore: European system for cardiac operative risk evaluation
ICU: intensive care unit
LVEF: Left ventricular ejection fraction
NYHA: New York heart association functional classification
NIRS 2002: Nutritional risk screening 2002
STS: Society of thoracic surgeons
VAS: Visual analog scale for pain assessment
